# Phase Transition Thermodynamics of 1,3,5-Tris-(α-naphthyl)benzene: Theory and Experiment

**DOI:** 10.3390/molecules29102180

**Published:** 2024-05-07

**Authors:** Mikhail I. Yagofarov, Dmitrii N. Bolmatenkov, Airat A. Notfullin, Andrey A. Sokolov, Ilya S. Balakhontsev, Timur A. Mukhametzyanov, Boris N. Solomonov

**Affiliations:** Department of Physical Chemistry, Kazan Federal University, Kremlevskaya Str. 18, 420008 Kazan, Russia; bolmatenkov@yandex.ru (D.N.B.); notfullinair@gmail.com (A.A.N.); andasokolov@kpfu.ru (A.A.S.); jsyoutub@gmail.com (I.S.B.); timur.mukhametzyanov@kpfu.ru (T.A.M.)

**Keywords:** vapor pressure, vaporization enthalpy, fusion enthalpy, solution calorimetry, heat capacity, polyaromatic hydrocarbons, fast scanning calorimetry, differential scanning calorimetry

## Abstract

1,3,5-Tris-(α-naphthyl)benzene is an organic non-electrolyte with notable stability of an amorphous phase. Its glassy and supercooled liquid states were previously studied by spectroscopic and calorimetric methods. Despite the continuing interest in its amorphous state and, particularly, vapor-deposited glasses, the thermodynamic parameters of the vaporization of 1,3,5-tris-(α-naphthyl)benzene have not been obtained yet. Likewise, the reliable evaluation of the thermodynamic parameters of fusion below the melting point, required to establish the thermodynamic state of its glass, is still an unsolved problem. In this work, the heat capacities of crystalline and liquid phases, the temperature dependence of the saturated vapor pressures, fusion and vaporization enthalpies were determined using differential and fast scanning calorimetry and were verified using the estimates based on solution calorimetry. The structural features of 1,3,5-tris-(α-naphthyl)benzene are discussed based on the computations performed and the data on the molecular refractivity. The consistency between the values obtained by independent techniques was demonstrated.

## 1. Introduction

Over the last 60 years, 1,3,5-tris-(α-naphthyl)benzene (C_36_H_24_, TNB) and its isomers proved to be important model objects in the studies of the glass transition phenomenon and crystallization [1,2,3,4,5,6,7]. TNB has a notably stable amorphous phase, so its glassy and supercooled liquid states can be investigated by a variety of spectroscopic and calorimetric methods. More recently, it was shown to form ultrastable glass upon physical vapor deposition onto a cold substrate [8,9,10,11].

The first detailed thermodynamic studies of 1,3,5-tris-naphthylbenzene were performed by Magill et al. [12,13,14]. In those works, the fusion enthalpy, solubility, vaporization enthalpy, crystallization kinetics and melt viscosity of TNB were reported. However, a comparison of the NMR spectrum of the specimen obtained by Magill et al. with the newly synthesized samples showed [6] that the isomer of TNB, 1,3-bis(1-naphthyl)-5-(2-naphthyl)benzene, has been studied in Refs. [12,13].

The fusion enthalpy at the melting point (*T*_m_) and the condensed phase heat capacities up to 370 K have been measured by Tsukushi et al. [2]. Describing the glass and supercooled liquid properties using modern approaches requires precise data on the thermodynamic parameters of fusion below *T*_m_ [15], so the determination of the heat capacities in the wider range is of critical importance, as is the validation of the results already present.

The thermodynamic parameters of vaporization are necessary for planning the vapor deposition process [16,17]. However, the extremely low volatility of TNB prevents studying crystal–vapor and liquid–vapor equilibria by conventional techniques.

To date, only the powder X-ray diffraction pattern of a TNB sample recrystallized from a chloroform–hexane mixture was described [1]. It is of interest to check whether different purification methods, including fractional sublimation, lead to the same crystal form.

**Figure 1:**
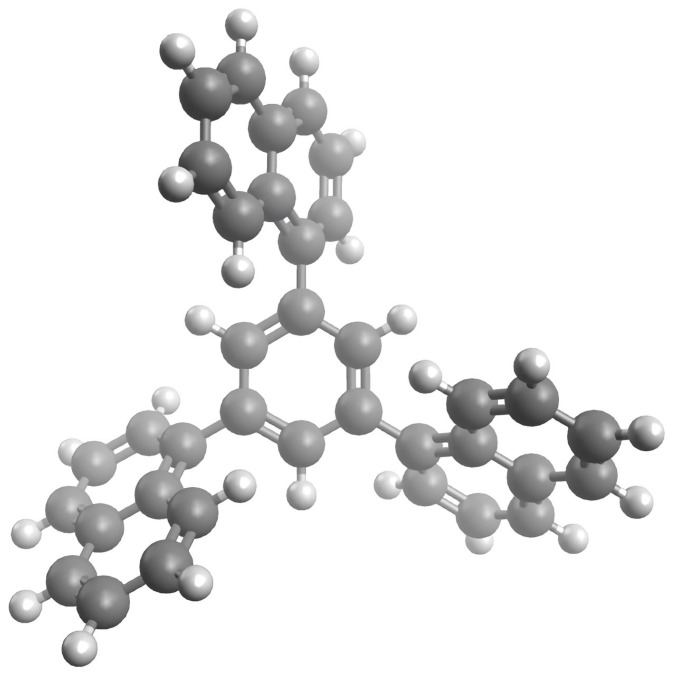
Optimized geometry of TNB in the gas phase obtained at the B3LYP/6-31+G(d,p) theory level.

The goal of the present work is to provide a consistent set of the thermodynamic parameters of the sublimation, fusion and vaporization of TNB as functions of temperature. Its fusion enthalpy and the heat capacities of crystal and liquid states were measured by differential scanning calorimetry (DSC), while the temperature dependence of the saturated vapor pressure and the vaporization enthalpy were determined by fast scanning calorimetry (FSC). The solution enthalpy in benzene was determined, and its agreement with the fusion enthalpy at 298.15 K calculated using Kirchhoff’s law of thermochemistry was demonstrated. The optimized geometry of the TNB molecule in the gaseous state (Figure 1), fundamental vibrational frequencies and the potential energy surface for the intramolecular rotations were computed, and the ideal gas phase heat capacities as a function of temperature were calculated, which, together with the measured liquid state heat capacity, enabled the establishment of the vaporization enthalpy at 298.15 K. On the other hand, the molecular refractivity of TNB was derived using refractometry and densitometry. This parameter was used to provide an estimate of the sublimation and vaporization enthalpies at 298.15 K, and it was found to agree with the experimental results.

## 2. Results

**Figure 2:**
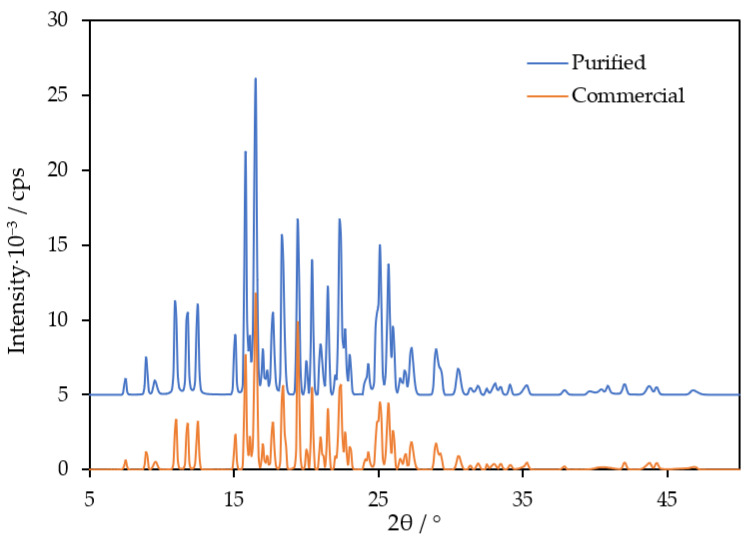
PXRD pattern of the commercial and purified TNB samples studied in this work.

Below, the results of the experimental measurements of the fusion and vaporization enthalpies, vapor pressures and heat capacities are provided together with the literature data. Before the measurement, TNB was purified by fractional sublimation under the reduced pressure of 10^−3^ Torr onto a substrate at *T* = 283 K. The vapor-deposited compound was mechanically removed from a sublimation apparatus, placed into a glass beaker and annealed at 443 K for 24 h in vacuo to ensure complete crystallization. Powder X-ray diffraction (PXRD) was employed to characterize the crystal structure (Figure 2; details of the experimental procedure may be found in the Supplementary Materials). The patterns were the same for the commercial and purified samples. The purity (mole fraction > 0.997) was determined using high-performance liquid chromatography. The information on the sample characterization is provided in Section 4.1 and Table S1.

### 2.1. Thermodynamics of Fusion

The fusion enthalpy at *T*_m_ ($\Delta_{\mathrm{cr}}^{l}H(T_{m})$) determined in this work equaled 32.8 ± 0.5 kJ·mol^−1^ (457.9 ± 0.3 K). The value corresponds to the average of the heating scans of the samples purified by vacuum sublimation, pre-melted and annealed at 443 K to 493 K at the rates of 1, 2, 5 and 10 K·min^−1^ (see Section 4.2). The uncertainties include the expanded uncertainty of the mean *U* (0.95 level of confidence, coverage factor of 2.0) and the reproducibility of calibration. The variation in the heating rate did not change $\Delta_{\mathrm{cr}}^{l}H$ or *T*_m_. The commercial samples initially melted with slightly lower enthalpy (28–31 kJ·mol^−1^), but after isothermal annealing at 443 K, the effect raised to ~33 kJ·mol^−1^.

The completely molten samples did not crystallize during further thermal cycling in DSC pans at (323–493) K at the rates from 1 to 30 K·min^−1^. The glass transition was observed at 353.0 ± 0.5 K (half-step glass transition temperature, *T*_g_) upon heating at 10 K·min^−1^ after cooling at 30 K·min^−1^. Tsukushi et al. [1] quenched the melt of TNB at ca. 300 K·min^−1^ and observed *T*_g_ = 355 K on heating at 3 K·min^−1^; in an adiabatic calorimetric study at the lower scanning rates, a *T*_g_ of 342 K was determined [2]. Whitaker and McMahon [6] reported *T*_g_ = 354 K at the heating rate of 20 K·min^−1^ but did not report the cooling rate.

Earlier, Tsukushi et al. [1] reported $\Delta_{\mathrm{cr}}^{l}H(T_{m})$ = 33.3 kJ·mol^−1^ (456.3 K), agreeing with the value determined in the present work. The authors [1] also performed a PXRD measurement of the crystal, which matches the pattern obtained in this work (Figure 2). It is worth noting that the melting points of TNB, available from the Reaxys database, vary between 428 and 476 K [18], suggesting polymorphism. In this work, both sublimed and commercial samples exhibited *T*_m_ values close to 458 K, agreeing with Refs. [1,6], where purification by recrystallization from organic solvents was performed. Excellent agreement between the *T*_m_ of the samples crystallized by different techniques, as well as the absence of other thermal events on the thermograms of TNB under various heating and scanning rates, rather suggest that the crystal form studied in this work and Refs. [1,6] is the most stable and easily producible. Variations of *T*_m_ [18] may be related to different isomers of TNB (the *T*_m_ of 1-(1-naphthyl)-3,5-bis(2-naphthyl)benzene equals 420 K; the *T*_m_ of 1,3-bis(1-naphthyl)-5-(2-naphthyl)benzene equals 467–472 K [6,12,13]).

The solution enthalpy of TNB in benzene was determined to independently evaluate the fusion enthalpy at 298.15 K (Table S4).

### 2.2. Condensed and Ideal Gas Phase Heat Capacities

**Figure 3:**
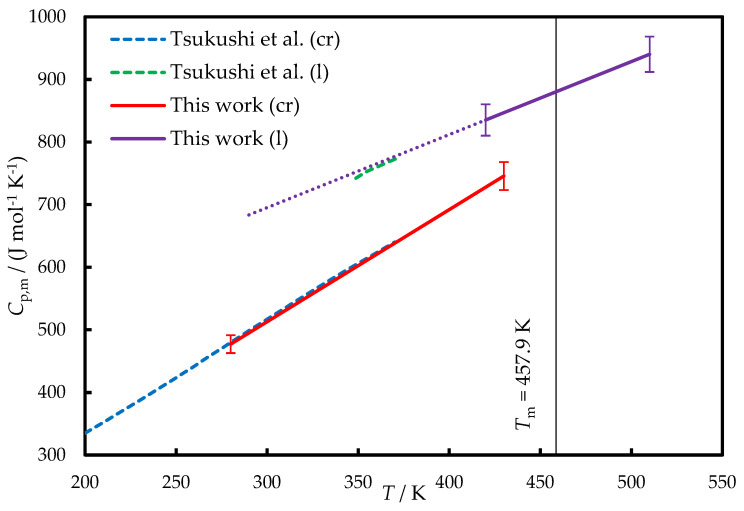
The heat capacities of TNB. Dashed blue line—crystal, Ref. [2]; solid red line—crystal, this work; dotted green line—liquid, Ref. [2]; solid violet line—liquid, this work. Dotted violet line—liquid, Equation (2). The smoothed experimental data obtained in this work are provided in Table S3.

The low crystallization tendency of TNB enables the determination of its heat capacity (*C*_p,m_) in the liquid state below *T*_m_ using conventional methods. The heat capacities of the liquid (l) and crystalline (cr) TNB measured in this work and by Tsukushi et al. [2] are provided in Figure 3.

In Ref. [2], Tsukushi et al. applied adiabatic calorimetry to measure the heat capacities of crystalline and glassy TNB; the latter measurements also included a narrow range of a supercooled liquid phase. For both crystal and liquid (in the case of linear extrapolation) phases, the agreement between our data and Ref. [2] is within ±1%. One can also compare these results with the crystal and liquid heat capacities of isomeric 1,3-bis(1-naphthyl)-5-(2-naphthyl)benzene, which were ~5% lower [12].

During the further calculation of the temperature dependences of the phase transition enthalpies, we used the *C*_p,m_ of crystal and liquid obtained in this work and fitted by the linear functions of temperature:

*C*_p,m_(cr)/(J mol^−1^ K^−1^) = 1.791 · (*T*/K) − 24.3, (280 < *T*/K < 430), *U*_r,tot_ < 0.03 (1)

*C*_p,m_(l)/(J mol^−1^ K^−1^) = 1.166 · (*T*/K) + 345.3, (420 < *T*/K < 510), *U*_r,tot_ < 0.03 (2)

*U*_r,tot_ is the expanded uncertainty (0.95 level of confidence), including the reproducibility of the measurement, calibration uncertainty and root-mean-square (*RMS*) of fitting (*RMS* and *R*^2^ equaled 0.7 J K^−1^ mol^−1^ and 0.999 for liquid and 2.3 J K^−1^ mol^−1^ and 0.997 for crystal).

The ideal gas phase heat capacities and entropies were calculated according to the procedure described in Section 4.5. The data necessary for *C*_p,m_(g) calculations are provided in Tables S5–S8. The resulting *C*_p,m_(g) values between 200 K and 800 K are presented in Table S8. The calculated ideal gas phase entropies are listed in Table S9.

### 2.3. Vapor Pressures and Vaporization Enthalpies

**Figure 4:**
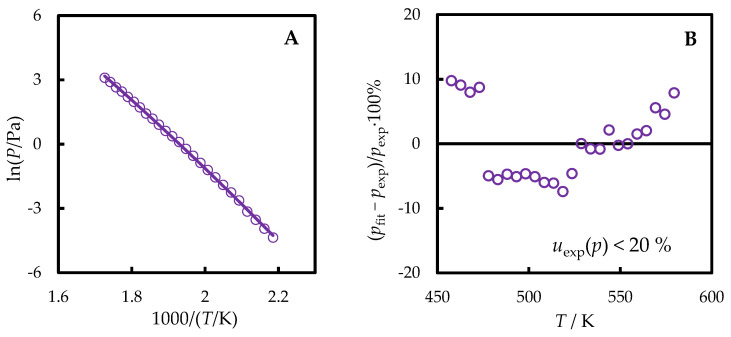
The vapor pressures of TNB measured in this work (violet line—values fitted by the Clarke–Glew equation, circles—experimental points) as functions of temperature (**A**) and the corresponding deviation plot from the dependence described by Equation (3) (**B**).

The vapor pressures (*p*) of TNB measured in this work are provided in Figure 4 and in Table 1.

The temperature dependence of the vapor pressure of TNB determined in this work was fitted by the Clarke–Glew equation:

(3) $$\begin{array}{c} \ln(p/\mathrm{Pa})=\ln(p(T_{\mathrm{ref}})/\mathrm{Pa})-\frac{\Delta_{l}^{g}H(T_{\mathrm{ref}})}{R}\left(\frac{1}{T}-\frac{1}{T_{\mathrm{ref}}}\right)\\ +\frac{\Delta_{l}^{g}C_{p,m}}{R}\left(\frac{T_{\mathrm{ref}}}{T}-1+\ln\left(\frac{T}{T_{\mathrm{ref}}}\right)\right) \end{array}$$

where the difference between the heat capacities of the ideal gas and liquid ($\Delta_{l}^{g}C_{p,m}$) was calculated based on the experimental (Section 2.2 and Section 4.3) *C*_p,m_(l) and computed (4.5) *C*_p,m_(g) values. In the temperature range of vapor pressure measurement, –$\Delta_{l}^{g}C_{p,m}$ non-monotonously varied between 154 and 164 J·K^−1^·mol^−1^, being 158 J·K^−1^·mol^−1^ on average. The middle of the measurement range was chosen as the reference temperature *T*_ref_ = 518 K. From the fitting, $\Delta_{l}^{g}H\left(518\;K\right)$ = 134.1 ± 3.2 kJ·mol^−1^ was derived (the standard uncertainty of the vaporization enthalpy, which includes the standard uncertainties of the vapor pressures and measurement temperatures, was evaluated as it was previously [20,21]).

One can compare the measured vapor pressures with the data on isomeric 1,3-bis(1-naphthyl)-5-(2-naphthyl)benzene [14]. Its saturated vapor pressures were determined at (703–803) K. The *p* values determined in this work and extrapolated to (703–803) K according to Equation (3) agreed with the data from Ref. [14] within ±50%. It should be mentioned that the thermal decomposition of 1,3-bis(1-naphthyl)-5-(2-naphthyl)benzene was reported to be above 723 K [14].

## 3. Discussion

In this section, the temperature dependences of the fusion and vaporization enthalpies are analyzed, and the consistency of the values at 298.15 K with the estimates from solution enthalpy in benzene and molecular refractivity is checked.

### 3.1. Fusion and Solution Thermochemistry at 298.15 K

In a series of our previous works [22,23,24], we showed that solution calorimetry enables the independent estimation of the fusion enthalpy at 298.15 K of organic non-electrolytes. In order to accomplish this, Hess’s law is applied to the solution process:

(4) $$\Delta_{\mathrm{cr}}^{l}H^{A}(298.15\;K)=\Delta_{\mathrm{soln}}H^{A/S}(cr,\;298.15\;K)-\Delta_{\mathrm{soln}}H^{A/S}(l,\;298.15\;K)\;$$

where $\Delta_{\mathrm{soln}}H^{A/S}(cr,\;298.15\;K)$ is the solution enthalpy of a crystal compound A in a solvent S at 298.15 K, and $\Delta_{\mathrm{soln}}H^{A/S}(l,\;298.15\;K)$ corresponds to the dissolution of a hypothetical quasi-equilibrium liquid A at 298.15 K, i.e., supercooled liquid obtained under equilibrium conditions. $\Delta_{\mathrm{soln}}H^{A/S}(cr,\;298.15\;K)$ is measured experimentally, while $\Delta_{\mathrm{soln}}H^{A/S}(l,\;298.15\;K)$ can often be estimated. It is usually close to 0 when A and S are structurally similar compounds (“like dissolves like”). Particularly, $\Delta_{\mathrm{soln}}H^{A/S}(l,\;298.15\;K)$ equals 1 ± 1 kJ·mol^−1^ when non-hydrogen-bonded aromatic compounds are dissolved in benzene [25]. In this work, $\Delta_{\mathrm{soln}}H^{{\mathrm{TNB}/C}_{6}H_{6}}(\mathrm{cr},\;298.15\;K)$ = 15.1 ± 0.4 kJ·mol^−1^ was determined (for details, see Section 4.2 and Table S4). From Equation (4), one can find $\Delta_{\mathrm{cr}}^{l}H(298.15\;K)$ = 14.1 ± 1.1 kJ·mol^−1^.

On the other hand, one can calculate the temperature dependence of the fusion enthalpy according to Kirchhoff’s law of thermochemistry. In the absence of phase transitions between 298.15 K and *T*_m_, Equation (5) is valid:

(5) $$\Delta_{\mathrm{cr}}^{l}H^{A}(298.15\;K)=\Delta_{\mathrm{cr}}^{l}H^{A}(T_{m})+\int_{T_{m}}^{298.15}[C_{p,m}^{A}(l)-C_{p,m}^{A}(\mathrm{cr})\;]dT$$

A linear temperature dependence of *C*_p,m_(l) between 298.15 K and *T*_m_ was assumed when calculating the $\int_{T_{m}}^{298.15}[C_{p,m}^{A}(l)-C_{p,m}^{A}(\mathrm{cr})\;]dT$ value. The validity of such an approximation was previously demonstrated for more than 50 compounds [22,23,24,26]. Using the $\Delta_{\mathrm{cr}}^{l}H(T_{m})$ = 32.8 ± 0.5 kJ·mol^−1^ determined in this work and the heat capacities given by Equations (1) and (2), one arrives at $\Delta_{\mathrm{cr}}^{l}H(298.15\;K)$ = (32.8 ± 0.5) − (21.4 ± 4.4) kJ·mol^−1^ = 11.5 ± 4.4 kJ·mol^−1^.

The $\Delta_{\mathrm{cr}}^{l}H(298.15\;K)$ values found according to Equations (4) and (5) agree within the limits of the propagated uncertainty, confirming the reliability of each experimental magnitude in the combined Hess’s and Kirchhoff’s law:

(6) $$\begin{array}{c} \Delta_{\mathrm{cr}}^{l}H^{A}(298.15\;K)=\Delta_{\mathrm{cr}}^{l}H^{A}(T_{m})+\int_{T_{m}}^{298.15\;K}[C_{p,m}^{A}(l)-C_{p,m}^{A}(\mathrm{cr})\;]dT\\ =\Delta_{\mathrm{soln}}H^{A/S}(cr,\;298.15\;K)-\Delta_{\mathrm{soln}}H^{A/S}(l,\;298.15\;K)\; \end{array}$$

The measured $\Delta_{\mathrm{cr}}^{l}H(T_{m})$ value and heat capacity temperature dependences given by Equations (1) and (2) can be implemented for estimating the thermodynamic stability of the supercooled liquid, as well as various glassy phases of TNB, which can be obtained by melt quenching, crystal milling or vapor deposition, with respect to this crystal form, and the parametrization of the kinetic parameters of crystallization/nucleation [15].

### 3.2. Vaporization and Sublimation Thermochemistry

Kirchhoff’s law of thermochemistry for the vaporization process (Equation (7)) was applied to calculate $\Delta_{l}^{g}H(298.15\;K)$:

(7) $$\Delta_{\mathrm{cr}}^{l}H(T_{2})=\Delta_{\mathrm{cr}}^{l}H(T_{1})+\int_{T_{1}}^{T_{2}}\Delta_{l}^{g}C_{p,m}dT$$

The *C*_p,m_(l) at *T* = (298.15–518.00) K was described by Equation (2). The *C*_p,m_(g) values (Table S8) were fitted to a quadratic polynomial. The $\Delta_{l}^{g}H(298.15\;K)$ value equaled (134.1 ± 3.2) + (38.5 ± 5.6) = 172.6 ± 6.4 kJ·mol^−1^. One can compare the latter with the estimates based on “molecular additivity” [27] and the correlation between the solvation enthalpy (enthalpy of the transition from the ideal gas to an infinitely diluted solution, $\Delta_{\mathrm{solv}}H$) and the molecular refraction (*MR*) [28].

First, it is fruitful to compare the vaporization enthalpies of 1-phenylnaphthalene and TNB. Previously, the average literature $\Delta_{l}^{g}H(298.15\;K)$ = 81.9 ± 1.0 kJ·mol^−1^ was derived for 1-phenylnaphthalene [28]. Then, the $\Delta_{l}^{g}H(298.15\;K)$ of TNB can be evaluated as (81.9 − 34.8) · 3 + 34.8 kJ·mol^−1^ = 176.1 kJ·mol^−1^ (34.8 kJ·mol^−1^ corresponds to the $\Delta_{l}^{g}H(298.15\;K)$ value of benzene accepted for the vaporization enthalpy calculation in Ref. [27]). It agrees with the experimental value within the measurement uncertainty. Such an estimate implies that phenyl and naphthalene rings exhibit the same conjugation between isolated aromatic fragments in 1-phenylnaphthalene and TNB. Our computations show that the phenyl-naphthyl dihedral angle in the optimized molecular structure of TNB equals (60.2 ± 0.2)°. In 1-phenylnaphthalene, the same angle equals (58 ± 4)° [29]. The potential energy surfaces also qualitatively agree (see Figure S1 for the potential surface computed for TNB), suggesting a similar level of conjugation in these molecules.

Molecular refractivity is a measure of molecule polarizability. Previously, it was shown that the $\Delta_{\mathrm{solv}}H(298.15\;K)$ values of organic non-electrolytes in various organic solvents correlate linearly with *MR*. Particularly, for aromatic hydrocarbons dissolved in benzene, Equation (8) is valid (root-mean-square deviation 0.8 kJ·mol^−1^ [28]):

(8) $$-\Delta_{\mathrm{solv}}H^{{A/C}_{6}H_{6}}(298.15\;K)/(\mathrm{kJ}\cdot {\mathrm{mol}}^{-1})=1.088\cdot \mathrm{MR}/({\mathrm{cm}}^{3}\cdot {\mathrm{mol}}^{-1})+6.86$$

In turn, the sublimation and vaporization enthalpies at 298.15 K can be found as the difference between the solution and solvation enthalpies:

(9) $$\Delta_{\mathrm{cr}/l}^{g}H^{A}(298.15\;K)=\Delta_{\mathrm{soln}}H^{{A/C}_{6}H_{6}}(\mathrm{cr}/l,\;298.15\;K)-\Delta_{\mathrm{solv}}H^{{A/C}_{6}H_{6}}(298.15\;K)\;$$

*MR* = 154.5 ± 3.2 cm^3^·mol^−1^ was calculated from the densities (*d*) and refractive indices (*n*) of TNB solutions in benzene measured in this work (Table S10). The *MR* of a pure compound can be found according to Equation (10) (*M* is a molecular weight):

(10) $$MR=\frac{M}{d}\left(\frac{n^{2}-1}{n^{2}+2}\right)$$

The *MR* of a solute is found according to Equation (11), considering that the molecular refractivities of solution components are additive:

(11) $$MR=\frac{1}{x}\left[\frac{M\cdot x+M^{C_{6}H_{6}}\cdot (1-x)}{d}\left(\frac{n^{2}-1}{n^{2}+2}\right)-MR^{C_{6}H_{6}}\cdot (1-x)\right]$$

where *x* and *M* correspond to the molar fraction and molecular weight of a solute.

From the literature, an *MR* of 160.0 cm^3^·mol^−1^ is available for TNB [30]. However, both the refractometer and densimeter used in Ref. [30] had an accuracy lower by an order of magnitude compared to the present work. Therefore, *MR* = 154.5 ± 3.2 cm^3^·mol^−1^ was used in the further calculations. From Equation (8), one can find $\Delta_{\mathrm{solv}}H^{{A/C}_{6}H_{6}}(298.15\;K)$ = –175.0 ± 3.6 kJ·mol^−1^. From this value, $\Delta_{\mathrm{cr}}^{g}H(298.15\;K)$ = 190.1 ± 3.6 kJ·mol^−1^ can be found using the experimental $\Delta_{\mathrm{soln}}H^{{A/C}_{6}H_{6}}(\mathrm{cr},\;298.15\;K)$ = 15.1 ± 0.4 kJ·mol^−1^. On the other hand, using an average estimate of $\Delta_{\mathrm{soln}}H^{{A/C}_{6}H_{6}}(l,\;298.15\;K)$ = 1 ± 1 kJ·mol^−1^ for non-hydrogen-bonded aromatic compounds, one can calculate $\Delta_{l}^{g}H(298.15\;K)$ = 176.0 ± 3.7 kJ·mol^−1^. The latter value agrees with the experimental data and the estimate based on the “molecular additivity” approach.

A comparison of the *MR* of 1-phenylnaphthalene (69.1 ± 1.0 cm^3^·mol^−1^ [28]) and TNB (154.5 ± 3.2 cm^3^·mol^−1^) with group-contribution-derived values is also useful. From the *MR* of benzene (26.18 cm^3^·mol^−1^), naphthalene (44.37 cm^3^·mol^−1^) and bond-refraction data (C-C, C-H) [30], the *MR*(calc) of 1-phenylnaphthalene equals 68.5 cm^3^·mol^−1^, indicating weak conjugation between the phenyl and naphthyl rings in this molecule. The *MR*(calc) of TNB would be 153.1 cm^3^·mol^−1^, which agrees with the experimental *MR* obtained in this work. Thus, it is likely that both TNB and 1-phenylnaphthalene exhibit weak conjugation between naphthyl and phenyl fragments. It is consistent with the observed vaporization enthalpies, which also reflect the polarizability of a molecule, and the dihedral phenyl-naphthyl angles of nearly 60°.

## 4. Materials and Methods

### 4.1. Materials

TNB (1,3,5-tris(1-naphthyl)benzene, C_36_H_24_, CAS No. 7059-70-3, Hotspot Biotechnology, Weifang, China) was purchased and purified by sublimation under reduced pressure. The final purity of 0.997 (mole fraction) was determined by HPLC using a Dionex Ultimate 3000 chromatograph (Thermo Fisher Scientific, Waltham, MA, USA) equipped with a UV detector (254 nm) and a Dionex Acclaim 120 chromatographic column (C18-bonded silica, 5 μm, 120 Å, 4.6 mm × 250 mm), and 100% acetonitrile was used as an eluent. The benzene used for solution calorimetry (C_6_H_6_, CAS No. 71-43-2) was purified according to [31]. Its final purity determined by gas chromatography (Agilent 7890 B, Santa Clara, CA, USA) exceeded 0.999 (mass fraction). The characterization of the samples and auxiliary compounds used for the calibration of the instruments is provided in Table S1.

### 4.2. Differential Scanning Calorimetry

The heat capacities, fusion enthalpy and melting temperature of TNB were measured using a DSC 8500 calorimeter (Perkin Elmer, Waltham, MA, USA).

The heat capacities were measured using a three-step method, with measurements taken for empty crucibles, sapphire and the sample. The temperature program involves two isothermal segments of 3 min at *T*_min_ and *T*_max_ and one dynamic segment at the rate of 10 K·min^−1^. For the crystalline phase, the measurements were performed between *T*_min_ = 280 and *T*_max_ = 430 K, while for the liquid phase, the range was 420 to 510 K. The heat capacities of the liquid determined below *T*_m_ correspond to the supercooled phase obtained by cooling the melt at a rate of 40 K/min. No crystallization or other effects were observed on the heating/cooling heat flow curves. Measurements of the heat capacity were repeated for three samples.

Initially, the melting point of 458 K was evaluated from the first heating scans of the purified TNB samples at 10 K·min^−1^. To ensure the reproducible thermal contact between the compound and the pan, the next samples were heated to 459 K with the onset of the melting process being observed, cooled to 443 K and annealed for 1 h. Their fusion enthalpies were determined during the heating scan between (*T*_m_ − 20 K) and (*T*_m_ + 20 K) at the rates of 1, 2, 5 and 10 K·min^−1^. Eight samples (two for each heating rate) were studied in total. The fusion enthalpies of the untreated and pre-melted samples are in agreement (Table S2).

The DSC was calibrated according to the manufacturer’s recommendations using zinc and indium samples. The verification of the quality of the DSC used in the heat capacity measurements with standard samples was performed previously [32]. All experiments were performed in 40 mL aluminum crucibles in a dynamic nitrogen atmosphere (Nurgas, Kazan, Russia; volume fraction of nitrogen > 0.99999) with a gas flow rate of 30 mL min^−1^. The experimental values are provided in Tables S2 and S3 of the Supplementary Materials.

### 4.3. Solution Calorimetry

The solution enthalpy of TNB in benzene was measured using an isothermal precision dissolution calorimeter TAM III (TA Instruments, New Castle, DE, USA). The measuring procedure was as follows: the crystalline samples with masses of ca. 40 mg were placed in glass ampoules. Subsequently, the ampoules were immersed in a calorimetric cell filled with 100 mL of benzene. Thereafter, the solution enthalpy was determined after the ampoule was broken in the pre-thermostated cells. Before and after the breaking of the ampoule, electrical calibrations were performed. Infinite dilution conditions were confirmed by monitoring the concentration dependence of the solution enthalpy (molality ranged from 1 to 6.5 mmol·kg^−1^). The accuracy of the technique was verified by the measurement of the solution enthalpy of 1-propanol in bidistilled water [33]. The obtained values of the solution enthalpies are provided in Table S4 of the Supplementary Materials.

### 4.4. Fast Scanning Calorimetry

The vapor pressures of TNB were measured using the thermogravimetry–fast scanning calorimetry method based on the relationship between the evaporation rate and vapor pressure [19], as previously described [21], using Flash DSC1 (Mettler Toledo, Greifensee, Switzerland). The UFS1 chip sensor was calibrated using anthracene, benzoic acid and biphenyl as temperature standards [34]. The measurement was performed between 455 K and 580 K under a nitrogen atmosphere (Nurgas, Kazan, Russia; volume fraction of nitrogen >0.99999) using samples with a typical mass of 50 ng in liquid and supercooled liquid states. No tendency to crystallize was observed during the measurements. The mass change during isothermal steps was calculated using the *C*_p,m_(liq) measured in this work by DSC. The sample geometry (the height and radius of the droplet) was determined using an optical microscope BX3M (Olympus, Tokyo, Japan). The diffusion volume of TNB needed to estimate its diffusion coefficient according to procedure [35] was calculated additively [36] and found to be 536.4 cm^3^ mol^−1^.

Measurements at each temperature were reproduced 10 times. The experimental values of the measured vapor pressures are provided in Table 1.

### 4.5. Computations

The heat capacities and entropies of TNB in the ideal gas phase between 200 and 800 K were calculated using methods of quantum chemistry and statistical thermodynamics.

First, the torsion angles between the benzene ring and naphthyl substituents were determined during a combined low-level/high-level conformational search algorithm implemented in the TorsiFlex v2022.1 software package [37]. A low-level search was conducted at the HF/3-21G theory level with the use of preconditioned and stochastic strategies. The preconditioned structures were made by a 30-degree rotation of the naphthyl substituents. Then, the high-level search was performed at the B3LYP/6-31+G(d,p) theory level, which is also used for all the subsequent computations in this work. The optimized geometry of TNB is shown in Figure 1; its cartesian coordinates are provided in Table S5. The correspondence of this structure to the global minimum was subsequently confirmed by the absence of negative vibrational frequencies and lower energy structures formed by internal rotation.

After that, a set of fundamental vibrational frequencies of TNB was obtained. The frequencies corresponding to internal rotation were identified with a method proposed by Ayala [38] and excluded from further calculations. All the other frequencies were scaled by a factor of 0.9795 below 2000 cm^−1^ and 0.9566 above 2000 cm^−1^, following the recommendations from Ref. [39]. The resulting set of frequencies was used for the calculation of the vibrational contribution to the ideal gas heat capacity and entropy according to Equations (S1) and (S4).

The one-dimensional (1-D) hindered rotor model was utilized for the treatment of the internal rotation motion to the heat capacities of the ideal gas phase. The algorithm described in Ref. [40] was used for this purpose, with the reduced moment of inertia of the naphthyl rotating top, *I*_r_ = 431.56 ± 0.14 amu Å^2^, calculated according to Kilpatrick and Pitzer [41]. For the determination of the potential energy surface (PES), a 360-degree optimized scan was performed with a step size of 10°. The obtained PES was used for the calculation of the energy levels of internal rotation with the Fourier grid Hamiltonian (FGH) method [42,43,44]. The contribution of hindered rotation to the ideal gas heat capacity and entropy of TNB is calculated from these energy levels with Equation (S2) and (S5) [40].

The implementation of the 1-DHR approach for the internal rotor treatment in combination with two scaling factors for vibrational frequencies allows for the determination of the ideal gas heat capacity and entropy with an average absolute percentage deviation *σ*_r_ of 1.5% at 300 K and 1% at 800 K [45].

All the data used for the determination of the ideal gas heat capacities and entropies of TNB are presented in Tables S5–S9 and Equations (S1)–(S8) of the Supplementary Materials. The calculated ideal gas heat capacity and entropy values between 200 and 800 K are presented in Tables S8 and S9.

### 4.6. Molecular Refractivity

For the determination of the molar refractivity value of TNB, the densities and refractive indices of its solutions in benzene were measured at 298.15 K.

An automatic digital refractometer RX-9000 alpha (Atago Co., Ltd., Tokyo, Japan) calibrated with de-ionized water was used for the determination of refractive indices of the TNB solutions in benzene at 298.15 K with an accuracy of 0.00010 in the range of measurements.

The densities of the benzene solutions of TNB at 298.15 K were measured using a vibrating-tube densimeter DSA 5000 M (Anton Paar, Graz, Austria) with an accuracy of 0.000007 g∙cm^−3^. Prior to the experiments, it was calibrated with air and deionized water.

The obtained refractive indices and densities of benzene solutions of TNB, as well as the calculated values of molar refractivity, are presented in Table S10 of the Supplementary Materials.

## Figures and Tables

**Table 1:** The vapor pressures above the liquid TNB measured in this work.

*T*/K	*p* ^a^/Pa	*T*/K	*p* ^a^/Pa	*T*/K	*p* ^a^/Pa
457.6	1.29 × 10^−2^	503.3	4.19 × 10^−1^	548.9	5.59
462.7	1.95 × 10^−2^	508.3	5.85 × 10^−1^	554.0	7.22
467.8	2.95 × 10^−2^	513.4	8.03 × 10^−1^	559.0	9.15
472.8	4.33 × 10^−2^	518.5	1.11	564.1	11.6
477.9	7.24 × 10^−2^	523.5	1.45	569.2	14.3
483.0	1.05 × 10^−1^	528.6	1.86	574.3	18.3
488.1	1.50 × 10^−1^	533.7	2.49	579.3	22.2
493.1	2.14 × 10^−1^	538.8	3.29		
498.2	2.99 × 10^−1^	543.8	4.19		

^a^ The estimated uncertainties (standard uncertainty (*u*) at a level of confidence of 68%) of vapor pressure reported in Table 1 were below 20% and included the uncertainty of sample mass determination, the sample area determination, the uncertainty of the diffusion coefficient and the uncertainty of temperature (1 K). An analysis of the uncertainties was made as described in Ref. [19].

## Data Availability

The original contributions presented in the study are included in the article and Supplementary Materials; further inquiries can be directed to the corresponding authors.

## Supplementary Materials

The following supporting information can be downloaded at: https://www.mdpi.com/article/10.3390/molecules29102180/s1, Figure S1: Potential energy surface for the internal rotation of naphthyl substituents in TNB; Table S1: Provenance and purity of the materials; Table S2: Enthalpies and temperatures of the fusion of TNB measured in this work at 0.1 MPa; Table S3: Isobaric heat capacities of crystalline and liquid TNB measured in this work using DSC at the pressure of 0.1 MPa; Table S4: Experimental enthalpies of the solution of TNB in benzene measured in this work at 298.15 K and 0.1 MPa; Table S5: Cartesian coordinates for TNB optimized with B3LYP/6-31+G(d,p); Table S6: Computed fundamental vibrational wavenumbers of TNB used in the calculation of the ideal-gas heat capacities; Table S7: Energy levels of the hindered rotation of naphthyl substituents in TNB; Table S8: Contributions of vibration and internal rotation to the heat capacities of TNB, as well as the isochoric and isobaric heat capacities calculated in this work; Table S9: Contributions of vibration and internal rotation to the entropies of TNB and its entropies in the ideal gas phase; Table S10: Molar refractions of TNB derived according to Equation (10) of the manuscript and experimentally measured densities and refractive indices of TNB solutions in benzene at 298.15 K required for its calculation [19,38,40,46].
