# Supplementary material for: Phase Transition Thermodynamics of 1,3,5-Tris-(α-naphthyl)benzene: Theory and Experiment
Source: Molecules. 2024 May 7;29(10):2180. doi: 10.3390/molecules29102180 (PMC11124026; doi:10.3390/molecules29102180)
Supplement: Supplementary file 1 [file molecules-29-02180-s001.zip › molecules-2965505-supplementary.pdf]

Supplementary Material

Phase Transition Thermodynamics of 1,3,5-tris-(α-naphthyl)benzene: theory and experiment

Mikhail I. Yagofarov[1], Dmitrii N. Bolmatenkov, Airat A. Notfullin, Andrey A. Sokolov, Ilya S. Balakhontsev, Timur A. Mukhametzyanov, and Boris N. Solomonov[2]

Department of Physical Chemistry, Kazan Federal University, Kremlevskaya str. 18, 420008 Kazan, Russia

1 Materials

Table S1
Provenance and purity of the materials.

Chemical Name	CASRN	Source	Sample purity	Purification method
TNB	15546-43-7	Hotspot Biotechnolog y	>0.98 [a]<br>>0.997 [c]	sublimation
biphenyl	92-52-4	Aldrich	0.999 [c]	none
benzoic acid	65-85-0	Aldrich	0.999 [c]	none
anthracene	120-12-7	Aldrich	0.999 [c]	none
indium	7440-74-6	PerkinElmer	0.99999 [a]	none
zinc	7440-66-6	PerkinElmer	0.99999 [a]	none
sapphire[d]	1344-28-1	PerkinElmer	not provided	none

[a] Initial purity stated by supplier;
[b] Purity after fractional sublimation *in vacuo* determined by HPLC (mole fraction);
[c] Purity determined by GC (mass fraction);
[d] Sapphire disc provided by Perkin Elmer (USA) as a reference material for the heat capacity measurements.

[1] To whom correspondence should be addressed: M. I. Yagofarov, MiIYagofarov@kpfu.ru
[2] To whom correspondence should be addressed: B. N. Solomonov, boris.solomonov@kpfu.ru

2 Differential scanning calorimetry

Table S2

Enthalpies and temperatures of fusion of TNP measured in this work at 10^5 Pa.

Sample mass / mg	T_m / K	$\Delta_{cr}^{l}H(T_m)$ / kJ mol^{-1}
Pre-melted samples		
Heating rate 1 K min^{-1}		
7.32	457.6	33.21
6.91	457.7	32.85
Heating rate 2 K min^{-1}		
6.43	457.8	32.33
6.85	457.7	32.92
Heating rate 5 K min^{-1}		
5.45	458.0	32.74
4.98	457.8	32.61
Heating rate 10 K min^{-1}		
4.21	458.2	33.10
3.89	458.2	32.35
**Average**	**457.9±0.3** [a]	**32.8±0.5** [a]
Non-pre-melted samples, heating rate 10 K min^{-1}		
4.14	458.2	33.19
4.71	459.0	33.12
4.88	458.5	32.29

[a] The uncertainties reported inside Table S2 include the expanded uncertainty of the mean *U* (0.95 level of confidence, coverage factor of 2.0) and reproducibility of calibration (0.1 K for temperature and 1 % for enthalpy); the standard uncertainty of pressure $u(p) = 5\cdot10^3$ Pa.

Table S3

Isobaric heat capacities of crystalline and liquid TNB measured in this work using DSC at the pressure of 10^5 Pa.

T / K	$C_{p,m}$ / J K^{-1} mol^{-1} [a]	$u(C_{p,m})$ / J K^{-1} mol^{-1}	T / K	$C_{p,m}$ / J K^{-1} mol^{-1} [a]	$u(C_{p,m})$ / J K^{-1} mol^{-1}
TNB (cr)			TNB (l)		
280	477.1	14.3	420	835.2	25.1
290	495.0	14.8	430	846.8	25.4
300	512.9	15.4	440	858.5	25.8
310	530.8	15.9	450	870.2	26.1
320	548.7	16.5	460	881.8	26.5
330	566.6	17.0	470	893.5	26.8
340	584.5	17.5	480	905.1	27.2
350	602.4	18.1	490	916.8	27.5
360	620.3	18.6	500	928.5	27.9
370	638.2	19.1	510	940.1	28.2
380	656.2	19.7			
390	674.1	20.2			
400	692.0	20.8			
410	709.9	21.3			
420	727.8	21.8			
430	745.7	22.4			

[a] The uncertainties include the expanded uncertainty of the mean U (0.95 level of confidence, coverage factor of 2.0) and the uncertainty of calibration; the standard uncertainty of pressure $u(p) = 5 \cdot 10^3$ Pa. The expanded uncertainty of temperature is ±0.1 K (0.95 level of confidence, coverage factor of 2.0).

3 Solution calorimetry

Table S4

Experimental enthalpies of solution of TNB in benzene measured in this work at 298.15 K and 0.1 MPa [a].

Mass of sample / mg	Molality [b] / mmol·kg^{-1}	$\Delta_{soln}H^{A_i/S}$ [c] / kJ mol^{-1}
40.2	1.00	14.88
45.7	2.14	15.60
45.0	3.26	14.54
45.3	4.39	16.03
41.2	5.41	15.01
42.1	6.47	14.75
		$\Delta_{soln}H^{A/S}$ = 15.13 ± 0.46 kJ·mol^{-1} [d]

[a] Standard uncertainties u are u(T) = 0.01 K, u(p) = 5 kPa.

[b] Molality of the solute in solution after experiments. Standard uncertainty u is 0.01 mmol·kg^{-1}.

[c] The experimental value obtained on each sample dissolution, assumed to correspond to the solution enthalpies at the infinite dilution.

[d] Average enthalpy of solution. Uncertainties reported inside Table S4 correspond to the combined expanded uncertainties of the mean U and the calibration reproducibilities (0.1 %), 0.95 level of confidence, coverage factor 2.0.

4 Powder X-ray diffraction

X-ray powder diffraction (XRPD) pattern of TNB was determined using a MiniFlex 600 diffractometer (Rigaku) equipped with a D/teX Ultra detector as reported previously [2]. In this experiment, Cu Kα radiation (40 kV, 15 mA) was used and data were collected at room temperature in the range of 2θ from 3 to 50° with a step of 0.02° and exposure time at each point of 0.24 s without sample rotation.

PXRD patterns of commercial TNB and the sample after fractional sublimation *in vacuo* and annealing at 443 K are provided in Figure 1.

5 Quantum chemical calculations

Table S5

Cartesian coordinates for TNB optimized with B3LYP/6-31+G(d,p).

Atom	X	Y	Z
C	-3.548334	-1.246553	0.196101
C	-2.862686	-0.497731	-0.822091
C	-3.586717	0.012054	-1.888318
C	-4.978585	-0.206743	-2.013862
C	-5.650716	-0.942957	-1.065244
C	-4.960712	-1.474123	0.057952
C	-2.899668	-1.750605	1.359989
C	-3.595427	-2.460732	2.315663
C	-4.981786	-2.705353	2.162555
C	-5.645888	-2.217166	1.059245
C	-1.391354	-0.249413	-0.766400
C	-0.474501	-1.313580	-0.764042
C	0.907929	-1.083017	-0.764172
C	1.370963	0.243110	-0.765832
C	0.480122	1.325022	-0.767676
C	-0.899917	1.063059	-0.768172
C	1.858694	-2.232968	-0.817805
C	1.773778	-3.121663	-1.878053
C	2.659830	-4.217276	-2.002341
C	3.639710	-4.424076	-1.058535
C	3.760596	-3.553842	0.058537
C	2.856459	-2.445019	0.195673
C	1.001090	2.723224	-0.823307
C	1.808686	3.093992	-1.886948
C	2.316357	4.408337	-2.011690
C	2.012111	5.359421	-1.064870
C	1.202118	5.029126	0.055259
C	0.691536	3.692670	0.192501
C	4.753321	-3.768243	1.054898
C	4.849882	-2.942272	2.152666
C	3.944050	-1.864543	2.305086
C	2.974786	-1.624260	1.354033
C	0.897671	5.995293	1.054241
C	0.137220	5.666133	2.154268
C	-0.346136	4.343841	2.306327
C	-0.075211	3.384930	1.353046
H	-5.518921	-3.269453	2.919495
H	-3.076827	-2.831889	3.194941
H	-1.840759	-1.563963	1.497669

Atom	X	Y	Z
H	-6.712700	-2.388406	0.939165
H	-6.718305	-1.124409	-1.158765
H	-5.508482	0.201156	-2.869700
H	-3.065659	0.574295	-2.657909
H	-0.841739	-2.335469	-0.769653
H	2.439529	0.436069	-0.772902
H	-1.601292	1.891991	-0.777091
H	2.037374	2.360945	-2.655215
H	2.937937	4.662195	-2.865410
H	2.390357	6.374176	-1.157655
H	1.284716	7.004156	0.934994
H	-0.085652	6.414341	2.909377
H	-0.931663	4.081797	3.182872
H	-0.445401	2.375330	1.489960
H	1.021885	-2.957201	-2.644527
H	2.567146	-4.885448	-2.853572
H	4.330990	-5.257704	-1.151285
H	5.435303	-4.606410	0.935718
H	5.612119	-3.119665	2.905799
H	4.010664	-1.224437	3.180104
H	2.283745	-0.800449	1.491515

Table S6

Computed fundamental vibrational wavenumbers of TNB used in the calculation of the ideal-gas heat capacities. The frequencies corresponding to the internal rotation were identified according to Ayala [3] and excluded from the table and further calculations.

ν / cm^{-1}	ν / cm^{-1}	ν / cm^{-1}	ν / cm^{-1}	ν / cm^{-1}
32.5	526.9	906.8	1212.8	1594.9
38.3	558.6	913.1	1213.1	1604.7
38.8	558.9	919.4	1213.6	1606.6
80.8	586.5	920.5	1238.8	1606.8
83.4	618.0	948.0	1239.3	1632.3
84.0	618.1	951.4	1252.3	1632.6
133.8	625.5	951.7	1263.8	1632.7
134.2	625.6	953.0	1269.8	3036.4
168.7	651.9	967.4	1270.2	3036.9
173.9	658.4	967.5	1313.6	3036.9
174.5	658.7	968.5	1331.1	3039.6
179.5	662.7	978.4	1337.1	3039.6
201.1	720.8	978.7	1337.2	3039.6
229.7	730.4	979.2	1344.3	3048.4

ν / cm^{-1}	ν / cm^{-1}	ν / cm^{-1}	ν / cm^{-1}	ν / cm^{-1}
229.9	730.7	988.3	1370.6	3048.5
268.1	733.8	1009.0	1370.7	3048.8
268.5	733.8	1009.0	1371.3	3049.7
276.1	734.8	1026.4	1394.1	3049.9
329.3	776.0	1029.9	1394.5	3050.2
329.5	776.1	1030.2	1407.6	3057.3
359.7	776.6	1063.4	1422.5	3057.6
401.3	786.3	1069.5	1423.1	3058.8
401.7	787.9	1069.9	1443.3	3062.3
426.3	788.2	1123.1	1448.2	3062.6
431.3	797.4	1123.6	1448.4	3062.7
431.6	797.8	1125.0	1466.0	3063.8
459.3	798.6	1146.9	1466.1	3063.8
471.9	803.8	1147.1	1466.7	3064.0
471.9	860.0	1147.8	1519.1	3078.7
474.6	860.6	1162.0	1519.4	3078.8
502.4	863.5	1162.2	1519.7	3079.0
502.6	865.9	1163.2	1587.5	
518.0	866.3	1177.4	1587.6	
518.1	892.6	1177.7	1589.7	
519.6	906.0	1191.5	1594.6	

Table S7

Energy levels of hindered rotation of naphthyl substituents in TNB (all three naphthyl rotating tops are equivalent, therefore they have equal energy levels of hindered rotation).

ν / cm^{-1}	ν / cm^{-1}	ν / cm^{-1}	ν / cm^{-1}	ν / cm^{-1}
15.2	481.9	1075.3	1813.5	2690.3
20.7	483.8	1081.4	1828.1	2704.4
37.8	492.0	1089.2	1830.1	2721.2
38.7	494.7	1094.9	1843.0	2739.6
54.2	502.3	1103.1	1846.7	2754.1
56.6	505.7	1108.4	1858.1	2771.5
70.2	512.6	1117.1	1863.4	2798.2
74.1	516.7	1122.0	1873.2	2820.3
85.9	523.1	1131.2	1880.1	2823.7
90.6	527.8	1135.6	1888.2	2825.4
101.3	533.6	1145.3	1896.8	2859.3
106.7	539.0	1149.3	1903.4	2878.0
116.6	544.3	1159.5	1913.6	2889.1
121.1	550.3	1162.9	1918.5	2922.9
122.6	555.0	1173.9	1930.5	2933.2

ν / cm^{-1}	ν / cm^{-1}	ν / cm^{-1}	ν / cm^{-1}	ν / cm^{-1}
131.6	561.6	1176.7	1933.5	2934.2
137.9	565.8	1188.2	1947.3	2960.2
140.4	572.9	1190.5	1948.8	2988.7
146.2	576.7	1202.7	1964.0	2992.2
153.1	584.3	1204.3	1964.2	3033.5
158.6	587.6	1217.2	1979.1	3052.2
160.4	595.8	1218.1	1981.2	3056.6
167.8	598.7	1231.8	1994.4	3067.1
174.1	607.3	1232.1	1998.1	3108.9
175.9	609.8	1246.0	2009.6	3114.4
182.2	618.8	1246.5	2015.1	3126.5
187.2	621.0	1260.0	2024.8	3178.7
192.2	630.2	1261.2	2032.2	3186.1
196.3	632.3	1274.0	2040.3	3198.1
199.5	641.7	1276.0	2049.3	3245.2
199.8	643.7	1288.0	2055.5	3261.9
207.2	653.0	1290.9	2066.5	3264.7
209.8	655.1	1302.1	2070.8	3271.2
210.9	664.1	1305.8	2083.6	3314.2
215.0	666.6	1316.2	2086.3	3344.2
219.7	674.7	1320.8	2100.8	3345.4
221.3	678.2	1330.4	2101.5	3385.6
222.7	684.6	1335.9	2116.9	3419.8
228.2	689.8	1344.6	2118.1	3423.9
228.6	694.1	1351.0	2132.6	3459.5
233.1	701.6	1358.8	2135.4	3479.2
234.5	704.0	1366.2	2147.8	3493.9
239.3	713.4	1373.1	2152.8	3502.8
239.4	714.5	1381.5	2163.3	3536.0
243.7	725.2	1387.4	2170.2	3566.0
245.8	725.4	1396.8	2179.1	3579.0
247.2	736.4	1401.7	2187.6	3615.1
252.2	737.1	1412.2	2194.2	3631.6
252.6	747.5	1416.1	2205.2	3649.3
257.4	749.1	1427.6	2209.9	3689.4
259.7	758.8	1430.5	2222.8	3696.5
263.2	761.2	1443.1	2225.7	3701.4
267.1	770.2	1444.9	2240.3	3721.4
269.3	773.3	1458.7	2240.7	3762.0
274.8	781.7	1459.3	2256.8	3780.2
275.7	785.5	1473.8	2257.7	3803.6
282.3	793.3	1474.3	2272.5	3849.2
282.6	797.8	1488.3	2275.7	3865.7

ν / cm^{-1}	ν / cm^{-1}	ν / cm^{-1}	ν / cm^{-1}	ν / cm^{-1}
289.2	805.1	1489.9	2287.3	3896.6
290.7	810.1	1502.9	2293.8	3917.6
296.3	817.0	1505.7	2303.9	3947.9
299.0	822.4	1517.4	2310.7	3952.8
303.6	829.0	1521.4	2319.4	4001.0
307.4	834.9	1532.0	2328.5	4040.6
311.1	841.1	1537.3	2333.6	4058.8
316.0	847.3	1546.6	2347.9	4116.7
318.8	853.3	1553.2	2351.4	4118.7
324.8	859.9	1561.3	2364.2	4128.4
326.7	865.6	1569.1	2366.4	4184.3
333.8	872.5	1575.9	2379.4	4214.1
334.8	878.0	1585.1	2380.1	4252.5
342.9	885.2	1590.6	2399.5	4277.5
343.0	890.5	1601.1	2400.4	4297.5
351.3	897.9	1605.3	2413.8	4328.2
352.1	903.2	1617.2	2423.5	4361.2
359.8	910.6	1620.0	2425.4	4407.1
361.5	915.9	1633.3	2433.3	4413.5
368.5	923.5	1634.7	2448.6	4474.6
371.1	928.7	1649.5	2449.7	4496.2
377.3	936.3	1649.5	2462.9	4544.3
380.7	941.6	1664.3	2473.6	4590.1
386.2	949.3	1665.7	2481.1	4618.3
390.5	954.6	1679.1	2497.7	4698.0
395.2	962.3	1681.9	2499.3	4700.8
400.5	967.6	1693.9	2510.6	4788.5
404.4	975.3	1698.2	2515.2	4813.1
410.5	980.8	1708.7	2526.3	4887.4
413.7	988.4	1714.6	2532.1	4948.7
420.7	994.0	1723.6	2548.7	4992.9
423.1	1001.5	1731.0	2570.7	5095.6
430.9	1007.4	1738.5	2575.9	5114.4
432.6	1014.7	1747.4	2582.5	5245.6
441.3	1020.8	1753.3	2587.3	5278.2
442.3	1028.0	1763.9	2603.2	5401.6
451.8	1034.3	1768.2	2612.7	5483.4
452.0	1041.3	1780.4	2628.9	5573.0
461.9	1047.9	1783.2	2630.4	5787.6
462.4	1054.6	1796.9	2631.3	5818.5
471.8	1061.6	1798.1	2673.5	6033.5
473.1	1068.0	1813.1	2683.6	6427.8

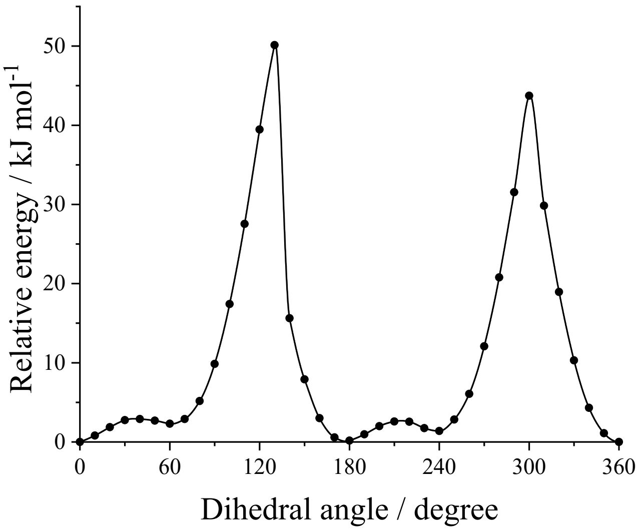

Fig. S1. Potential energy surface for internal rotation of naphthyl substituents in TNB. A dihedral angle of 0 degrees corresponds to the optimal configuration.

Table S8

Contributions of vibration and internal rotation to the heat capacities of TNB, as well as isochoric and isobaric heat capacities calculated in this work.

T / K	$C_{v.vib}(T)$ / J K^{-1} mol^{-1} [a]	$\sum C_{v.ir}(T)$ / J K^{-1} mol^{-1} [b]	$C_{v.m}(T)$ / J K^{-1} mol^{-1}	$C_{p.m}(T)$ / J K^{-1} mol^{-1}
200	254.5	24.0	303.4	311.7
220	287.6	22.9	335.4	343.7
240	321.5	22.1	368.6	376.9
260	356.0	21.5	402.4	410.7
280	390.6	20.9	436.5	444.8
298.15	421.9	20.5	467.3	475.7
300	425.0	20.5	470.5	478.8
320	459.1	20.1	504.2	512.5
340	492.4	19.9	537.2	545.5
360	525.0	19.6	569.5	577.8
380	556.5	19.4	600.9	609.2
400	587.0	19.2	631.1	639.5
420	616.4	19.0	660.3	668.6
440	644.6	18.9	688.4	696.7
460	671.6	18.7	715.2	723.5
480	697.4	18.6	740.9	749.3
500	722.1	18.4	765.5	773.8
520	745.7	18.3	789.0	797.3
540	768.3	18.2	811.4	819.7
560	789.8	18.0	832.8	841.1

T / K	$C_{v.vib}(T)$ / J K^{-1} mol^{-1} [a]	$\sum C_{v.ir}(T)$ / J K^{-1} mol^{-1} [b]	$C_{v.m}(T)$ / J K^{-1} mol^{-1}	$C_{p.m}(T)$ / J K^{-1} mol^{-1}
580	810.4	17.9	853.2	861.5
600	830.0	17.8	872.7	881.0
620	848.7	17.7	891.3	899.7
640	866.7	17.5	909.1	917.5
660	883.8	17.4	926.2	934.5
680	900.2	17.3	942.4	950.8
700	916.0	17.1	958.0	966.3
720	931.0	17.0	972.9	981.3
740	945.5	16.8	987.3	995.6
760	959.4	16.7	1001.0	1009.3
780	972.7	16.5	1014.1	1022.4
800	985.5	16.4	1026.8	1035.1

[a] Vibrational contribution to the heat capacity. Calculated based on the set of frequencies listed in Table S7 using Eq. (S1).
[b] Sum of the internal rotation contributions for all rotating tops of the molecule.

The procedure of ideal gas phase heat capacities calculation

According to the rigid rotor − harmonic oscillator model, the vibrational contribution to the molar heat capacity in the ideal gas phase can be calculated by Eq. (S1) with the set of frequencies from Table S6:

$$C_{\mathrm{v,vib}} = R \cdot \sum_i \frac{(\frac{\Theta_i}{T})^2 \cdot \exp(\frac{\Theta_i}{T})}{(\exp(\frac{\Theta_i}{T})-1)^2}, \tag{S1}$$

where Θ_i is the i-th fundamental vibrational frequency of the molecule.

The contribution of hindered rotation from each rotating top is calculated by Eq. (S2) [4] using the energy levels listed in Table S7:

$$C_{\mathrm{v,ir}} = \frac{\left(\sum_j \exp(-\frac{\varepsilon_j}{kT}) \cdot \sum_j \frac{\varepsilon_j^2}{kT^2} \exp(-\frac{\varepsilon_j}{kT})\right) - \left(\sum_j \frac{\varepsilon_j}{kT^2} \exp(-\frac{\varepsilon_j}{kT}) \cdot \sum_j \varepsilon_j \exp(-\frac{\varepsilon_j}{kT})\right)}{\left(\sum_j \exp(-\frac{\varepsilon_j}{kT})\right)^2}, \tag{S2}$$

where ε_j is the frequency of the j-th energy level.

The molar isobaric heat capacity in the ideal gas phase can be found by summing the contributions of vibration ($C_{\mathrm{v,vib}}$), internal rotation of all rotating tops ($\sum C_{\mathrm{v,ir}}$), translation (3/2 R), overall rotation (3/2 R), and the difference between isobaric and isochoric heat capacity (R):

$$C_{\mathrm{p,m}} = C_{\mathrm{v,vib}} + \sum C_{\mathrm{v,ir}} + C_{\mathrm{v,trn}} + C_{\mathrm{v,rot}} + (C_{\mathrm{p,m}} - C_{\mathrm{v,m}}) = C_{\mathrm{v,vib}} + \sum C_{\mathrm{v,ir}} + 4R \tag{S3}$$

Table S9

Contributions of vibration and internal rotation to the entropies of TNB, as well as entropies themselves calculated in this work.

T / K	$S_{vib}(T)$ / J K^{-1} mol^{-1} [a]	$\sum S_{ir}(T)$ / J K^{-1} mol^{-1} [b]	$S(T)$ / J K^{-1} mol^{-1}
200	210.3	110.3	755.6
220	236.1	112.5	786.8
240	262.6	114.5	818.2
260	289.7	116.2	849.7
280	317.3	117.8	881.3
298.15	342.8	119.1	910.2
300	345.4	119.2	913.2
320	374.0	120.5	945.2
340	402.8	121.7	977.2
360	431.9	122.9	1009.3
380	461.1	123.9	1041.4
400	490.4	124.9	1073.4
420	519.8	125.9	1105.4
440	549.1	126.7	1137.1
460	578.4	127.6	1168.7
480	607.5	128.4	1200.0
500	636.5	129.1	1231.1
520	665.3	129.8	1261.9
540	693.8	130.5	1292.4
560	722.2	131.2	1322.6
580	750.2	131.8	1352.5
600	778.1	132.4	1382.0
620	805.6	133.0	1411.2
640	832.8	133.6	1440.1
660	859.7	134.1	1468.6
680	886.4	134.6	1496.7
700	912.7	135.1	1524.5
720	938.7	135.6	1551.9
740	964.4	136.1	1579.0
760	989.8	136.5	1605.8
780	1014.9	136.9	1632.1
800	1039.7	137.3	1658.2

[a] Vibrational contribution to the entropy. Calculated based on the set of frequencies listed in Table S6 using Eq. (S4).

[b] Sum of the internal rotation contributions for all rotating tops of the molecule.

The procedure of ideal gas phase entropy calculation

According to the rigid rotor – harmonic oscillator model, the vibrational contribution to the entropy in the ideal gas phase can be calculated by Eq. (S4) with the set of frequencies from Table S6:

$$S_{\mathrm{vib}} = R \cdot \sum_i \left(\frac{\frac{\Theta_i}{T}}{\exp(\frac{\Theta_i}{T}) - 1} - \ln\left(1 - \exp(-\frac{\Theta_i}{T})\right) \right), \quad \text{(S4)}$$

where Θ_i is the i-th fundamental vibrational frequency of the molecule.

The contribution of hindered rotation from each rotating top is calculated by Eq. (S5) [4] using the energy levels listed in Table S7:

$$S_{\mathrm{ir}} = k \ln\left(\frac{1}{\sigma_{\mathrm{r}_i}} \sum_j \exp(-\frac{\varepsilon_j}{kT}) \right) + \frac{1}{T} \frac{\sum_j \varepsilon_j \exp(-\frac{\varepsilon_j}{kT})}{\sum_j \exp(-\frac{\varepsilon_j}{kT})}, \quad \text{(S5)}$$

where σ_{r_i} is the internal symmetry number of the i-th rotating top ($\sigma_{\mathrm{r}_i} = 1$ for all naphthyl rotating tops of TNB) and ε_j is the frequency of the j-th energy level.

The translational entropy of an ideal gas was found using Eq. S6 (M = 456.6 g/mol, $P = 10^5$ Pa):

$$S_{\mathrm{trn}} = R \ln\left[\frac{1}{N_{\mathrm{A}}} \cdot \left(\frac{2\pi MkT}{h^2 N_{\mathrm{A}}} \right)^{3/2} \cdot \frac{RT}{P} \right] + \frac{5}{2} R \quad \text{(S6)}$$

The rotational entropy of TNB was calculated using moments of inertia around principal axes ($J_1 = J_2 = 8.80 \cdot 10^{-40}$ kg·m^2, $J_3 = 1.44 \cdot 10^{-39}$ kg·m^2), the symmetry number of TNB ($\sigma = 3$) and Eq. S7:

$$S_{\mathrm{rot}} = R \ln\left[\frac{\sqrt{\pi}}{\sigma} \cdot \sqrt{J_1 J_2 J_3} \cdot \left(\frac{8\pi^2 kT}{h^2} \right)^{3/2} \right] + \frac{3}{2} R \quad \text{(S7)}$$

The entropy in the ideal gas phase can be found by summing the contributions of vibration (S_{vib}), internal rotation of all rotating tops ($\sum S_{\mathrm{ir}}$), translation (S_{trn}) and overall rotation (S_{rot}):

$$S = S_{\mathrm{vib}} + \sum S_{\mathrm{ir}} + S_{\mathrm{trn}} + S_{\mathrm{rot}} \quad \text{(S8)}$$

6 Molecular refractivity determination

Table S10

Molar refractions of TNB derived according to Eq. (10) of the manuscript and experimentally measured densities and refractive indices of TNB solutions in benzene at 298.15 K required for its calculation (uncertainties are expressed as standard deviation).

Compound	Mole fraction $\times 10^{3}$ [a]	Density [b] / $g \cdot cm^{-3}$	Refractive index [c]	*MR* / $cm^{3} \cdot mol^{-1}$
TNB	1.026	0.875156	1.49872	154.8
	1.700	0.876125	1.49931	152.5
	2.652	0.877482	1.50043	156.2
				Density (benzene) = 0.873666 $g \cdot cm^{-3}$
				Refractive index (benzene) = 1.49768
				***MR* = 154.5 ± 3.2 $cm^{3} \cdot mol^{-1}$ [d]**

[a] Mole fraction of TNB in benzene solutions. Standard uncertainty *u* is 0.002.
[b] Density of TNB solution in benzene at a given molality (accuracy stated by the device manufacturer is ±0.000007 $g \cdot cm^{-3}$).
[c] Refractive index of TNB solution in benzene at a given molality (accuracy stated by the device manufacturer is ±0.00010).
[d] The standard uncertainty of the mean *MR* value is the propagated error, including the reproducibility of experimental *MR* and uncertainties of the refractive index and density.

References

1. Buzyurov, A.V.; Nagrimanov, R.N.; Zaitsau, D.H.; Mukhametzyanov, T.A.; Solomonov, B.N.; Abdelaziz, A.; Schick, C. Application of the Flash DSC 1 and 2+ for vapor pressure determination above solids and liquids. *Thermochimica Acta* **2021**, *706*, 179067, doi:10.1016/j.tca.2021.179067.
2. Ziganshin, M.A.; Gerasimov, A.V.; Ziganshina, S.A.; Gubina, N.S.; Abdullina, G.R.; Klimovitskii, A.E.; Gorbatchuk, V.V.; Bukharaev, A.A. Thermally induced diphenylalanine cyclization in solid phase. *Journal of Thermal Analysis and Calorimetry* **2016**, *125*, 905-912.
3. Ayala, P.Y.; Schlegel, H.B. Identification and treatment of internal rotation in normal mode vibrational analysis. *The Journal of Chemical Physics* **1998**, *108*, 2314-2325.
4. Pfaendtner, J.; Yu, X.; Broadbelt, L.J. The 1-D hindered rotor approximation. *Theoretical Chemistry Accounts* **2007**, *118*, 881-898.
